# Risk factors for acute surgical site infections after lumbar surgery: a retrospective study

**DOI:** 10.1186/s13018-017-0612-1

**Published:** 2017-07-19

**Authors:** Qi Lai, Quanwei Song, Runsheng Guo, Haidi Bi, Xuqiang Liu, Xiaolong Yu, Jianghao Zhu, Min Dai, Bin Zhang

**Affiliations:** 1 Department of Orthopedics, Artificial Joints Engineering and Technology Research Center of Jiangxi Province, The First Affiliated Hospital of Nanchang University, 17 Yongwai Street, Nanchang, Jiangxi 330006 People’s Republic of China; 2 Department of Orthopedics, Multidisciplinary Therapy Center of Musculoskeletal Tumor, The First Affiliated Hospital of Nanchang University, Nangchang, 330006 Jiangxi China

**Keywords:** Postoperative infection, Lumbar surgery, Risk factor, Prevention, Strategy

## Abstract

**Background:**

Currently, many scholars are concerned about the treatment of postoperative infection; however, few have completed multivariate analyses to determine factors that contribute to the risk of infection. Therefore, we conducted a multivariate analysis of a retrospectively collected database to analyze the risk factors for acute surgical site infection following lumbar surgery, including fracture fixation, lumbar fusion, and minimally invasive lumbar surgery.

**Methods:**

We retrospectively reviewed data from patients who underwent lumbar surgery between 2014 and 2016, including lumbar fusion, internal fracture fixation, and minimally invasive surgery in our hospital’s spinal surgery unit. Patient demographics, procedures, and wound infection rates were analyzed using descriptive statistics, and risk factors were analyzed using logistic regression analyses.

**Results:**

Twenty-six patients (2.81%) experienced acute surgical site infection following lumbar surgery in our study. The patients’ mean body mass index, smoking history, operative time, blood loss, draining time, and drainage volume in the acute surgical site infection group were significantly different from those in the non-acute surgical site infection group (*p <* 0.05). Additionally, diabetes mellitus, chronic obstructive pulmonary disease, osteoporosis, preoperative antibiotics, type of disease, and operative type in the acute surgical site infection group were significantly different than those in the non-acute surgical site infection group (*p <* 0.05). Using binary logistic regression analyses, body mass index, smoking, diabetes mellitus, osteoporosis, preoperative antibiotics, fracture, operative type, operative time, blood loss, and drainage time were independent predictors of acute surgical site infection following lumbar surgery.

**Conclusions:**

In order to reduce the risk of infection following lumbar surgery, patients should be evaluated for the risk factors noted above.

**Electronic supplementary material:**

The online version of this article (doi:10.1186/s13018-017-0612-1) contains supplementary material, which is available to authorized users.

## Background

Acute surgical site infection (ASSI) following lumbar surgery is a serious complication with significant morbidity and economic burden. Despite the use of prophylactic antibiotics and improvements in surgical techniques and postoperative care, acute wound infections continue to affect patients after lumbar surgery [1–3]. Patients with ASSI have longer hospital stays, higher reoperation rates, and serious back pain [4]. Although China’s financial investment in healthcare is growing along with a corresponding increase in medical insurance, the medical costs for a patient with an ASSI after lumbar surgery have increased to be within the range of $ 0.5–2 million.

ASSI after lumbar surgery is a commonly reported complication. Studies from European populations report infection rates ranging from 9.3 to 20% [5, 6]. Although techniques for spinal surgery have improved with regard to postoperative infections and wound complications, the infection rates are still high. Additionally, although many scholars have published findings related to surgical wound infections, they did not perform a systematic assessment of the risk factors for ASSI following lumbar surgery. Unlike previous studies, our study had a large sample size, and we performed a comprehensive assessment of the risk factors of ASSI. Moreover, we focused on ASSI, which is significantly different from the SSI that was reported in other studies. The aim of our study was to analyze the risk factors for ASSI following lumbar surgery, including fracture fixation, lumbar fusion, and minimally invasive lumbar surgery in order to provide clinicians with a theoretical basis for preventing ASSI after lumbar surgery. Our goal was to help reduce the infection rate and the patients’ physical, mental, and economic burdens.

## Methods

After approval by our hospital’s ethics committee, we performed a review of all lumbar surgeries performed at the orthopedic department of The First Affiliated Hospital of Nanchang University to identify patients who developed an ASSI. All surgeries were performed by the director of spine surgery. Patients who underwent lumbar fusion, internal fracture fixation, and minimally invasive surgery between January 2014 and December 2016 were identified by searching the hospital’s medical record database. During this period, 1367 patients underwent lumbar surgery. Cases of lumbar vertebra fracture, lumbar intervertebral disc herniation, lumbar canal stenosis, degenerative lumbar spondylolisthesis, and scoliosis were considered in this analysis.

### Identification of acute surgical site infection

ASSIs as classified according to the criteria of the Centers for Disease Control and Prevention in China were studied. An infection was considered to be an ASSI when it occurred at the surgical site within 2 weeks after surgery. ASSI was defined as an infection involving the deep soft tissue muscle and fascia, in contrast to a superficial infection with only infected skin and subcutaneous tissue. Additional criteria for ASSI were the presence of at least one classical sign of inflammation (pain, swelling, redness, increased local temperature) and drainage of purulent fluid from the operative incision, spontaneous wound dehiscence, or an abscess or other signs of infection on observation, reoperation, or histopathological or radiological investigation [7, 8].

### Data collection

Patients were selected according to the following criteria: (1) a preoperative diagnosis of lumbar vertebra fracture, lumbar intervertebral disc herniation, lumbar canal stenosis, degenerative lumbar spondylolisthesis, or scoliosis and (2) patients with complete data who underwent lumbar fusion surgery. Patients were excluded if they had a primary lumbar infection, such as lumbar spine tuberculosis, or non-lumbar surgery; were less than 18 years old; were dependent on pain or psychotropic medications; or had cognitive or mental disorders. Each procedure was performed by board-certified spinal surgeons at a dedicated tertiary general hospital. Data review was then performed, and missing data of 444 patients were excluded.

Risk factors were analyzed, including patient-related risk factors such as sex, age, body mass index (BMI), educational level, type of disease, smoking, alcohol consumption, long-term hormone use, hypertension, chronic obstructive pulmonary disease (COPD), osteoporosis, diabetes, nutritional status, and procedure-related risk factors such as preoperative antibiotics, operative time, intraoperative blood loss, intraoperative blood transfusion, number of internal fixation metals, postoperative drainage time, and drainage volume.

### Statistical analysis

Twenty-six patients were found to have ASSI after lumbar surgery and were defined as the infection group; the other 897 patients were defined as the control group. The count data were analyzed by the chi-square test, and the measurement data were analyzed by an independent sample *t* test. Binary logistic regression controlled for confounding characteristics and identified independent predictors of postoperative surgical site infections. All the data were processed by SPSS 23.0 statistical software (IBM Corp., Armonk, NY). *p <* 0.05 was considered to be statistically significant.

## Results

Overall, 1367 patients were identified who met our inclusion criteria. A total of 368 patients with incomplete information because of transfer to another hospital and 76 patients who declined the operation on the day of surgery were excluded. Thus, 923 patients were included in this analysis. Twenty-six patients were diagnosed with lumbar ASSI. The incidence of ASSI was 2.81%.

Age, BMI, smoking history, number of internal fixation metals, operative time, blood loss, operative incision, drainage tube, drainage time, and drainage volume were the measurement data analyzed by the *t* test. The mean BMI, smoking history, operative time, blood loss, drainage time, and drainage volume in the ASSI group were significantly different from those in the non-ASSI group (*p <* 0.05, Table 1). Additionally, sex, alcohol intake, educational level, diabetes mellitus, COPD, osteoporosis, preoperative antibiotics, long-term hormone use, intraoperative blood transfusion, nutritional status, hypertension, type of disease, and operative type were analyzed as count data by the chi-square test. Diabetes mellitus, COPD, osteoporosis, preoperative antibiotics, type of disease, and operative type in the ASSI group were found to have statistically significant differences compared to the non-ASSI group (*p <* 0.05, Table 2). Finally, the risk factors for the statistical differences were analyzed by binary logistic regression. BMI, smoking, diabetes mellitus, osteoporosis, preoperative antibiotics, fracture, operative type, operative time, blood loss, and drainage time were independent predictors of ASSI following lumbar surgery (*p <* 0.05 and Exp (B) > 1, Table 3).

**Table 1:** The differences of risk factors in the infection and non-infection groups were analyzed by *t* test (measurement data)

Risk factors	ASSI (mean ± SEM, *n* = 26)	Non-ASSI (mean ± SEM, *n* = 897)	*t*	*p*
Age (year)	54.58 ± 2.710	54.93 ± 0.5503	0.1090	0.9132
BMI (kg/m^2^)	24.85 ± 0.9210	22.76 ± 0.1179	2.935	*0.0034*
Smoking (year)	21.27 ± 2.765	10.18 ± 0.3913	4.770	*<0.0001*
Number of internal fixation metals (piece)	5.423 ± 0.4374	5.252 ± 0.07764	0.3703	0.7112
Operative time (min)	208.5 ± 7.439	170.7 ± 2.776	2.307	*0.0212*
Blood loss (ml)	916.2 ± 72.66	696.5 ± 12.79	2.886	*0.0040*
Operative incision (cm)	13.38 ± 0.8340	11.93 ± 0.2319	1.061	0.2889
drainage tube (root)	1.538 ± 0.1385	1.394 ± 0.0306	0.7985	0.4248
Time of draining (day)	3.808 ± 0.3283	2.146 ± 0.05885	4.745	*<0.0001*
Capacity draining (ml)	440.0 ± 45.92	236.7 ± 8.072	4.230	*<0.0001*

Italicized value is statistically different

**Table 2:** The differences of risk factors in the infection and non-infection groups were analyzed by Chi-square test (count data)

Risk factors		ASSI (*n* = 26)	Non-ASSI (*n* = 897)	*χ* ^2^	*p*
Sex	Male	20	550	2.606	0.106
	Female	6	347		
Drink wine	Yes	19	550	1.478	0.224
	No	7	347		
Academic career	≤High school	24	770	0.897	0.349
	>High school	2	127		
Diabetes mellitus	Yes	17	382	5.351	*0.021*
	No	9	515		
COPD	Yes	10	563	6.340	*0.012*
	No	16	334		
Osteoporosis	Yes	19	476	4.069	*0.044*
	No	7	421		
Preopreative antibiotics	Yes	19	456	5.004	*0.025*
	No	7	441		
Type of disease	Fracture	15	308	6.059	*0.014*
	Others	11	589		
Operative type	Open	23	621	4.431	*0.035*
	Others	3	276		
Long-term use of hormone	Yes	6	98	3.732	0.053
	No	20	799		
Intraoperative blood transfusion	Yes	5	206	0.614	0.433
	No	21	567		
Nutritional status	Good	19	687	0.173	0.667
	Poor	7	210		
Hypertension	Yes	4	245	1.825	0.177
	No	22	652		

Italicized value is statistically different

**Table 3:** Binary logistic regression model for the development of ASSI after lumbar surgery

Risk factors		Exp (B) (95% C.I. of Exp (B))	*p*
Patient-related risk factors	Age (year)	0.729(0.544–0.976)	0.34
	Sex	0.000(0.000–2.294)	0.074
	BMI (kg/m^2^)	2.888(1.059–7.875)	*0.038*
	Smoking (year)	1.684(1.008–2.813)	*0.047*
	Drink wine	2.180(0.241–9.771)	0.121
	Academic career	3.337(0.012–9.383)	0.265
	Diabetes mellitus	2.200(0.046–1.102)	*0.020*
	COPD	0.000	0.987
	Osteoporosis	1.842(0.151–4.836)	*0.044*
	Nutritional status	0.000(0.000–162.412)	0.220
	Fracture	2.916(0.156–5.308)	*0.001*
	Hypertension	0.011(0.00–13.221)	0.213
	Long-term use of hormone	0.551(0.151–4.836)	0.105
Procedure-related risk factors	Preoperative antibiotics	2.030(0.005–5.216)	*0.025*
	Operative type	1.374(0.010–4.445)	*0.035*
	Operative incision (cm)	1.027(0.555–1.899)	0.993
	Operative time (min)	1.014(0.987–1.042)	*0.030*
	Blood loss (ml)	1.022(0.999–1.045)	*0.024*
	Number of internal fixation metals (piece)	22.589(0.891–572.990)	0.059
	Intraoperative blood transfusion	0.000(0.000–0.417)	0.413
	Drainage tube (root)	0.019(0.000–11.327)	0.225
	Time of draining (day)	4.983(1.641–15.140)	*0.033*
	Capacity draining (ml)	1.008(0.990–1.025)	0.392

DM, osteoporosis, COPD, preoperative antibiotics, fracture and operative type are classification variables; BMI, smoking, operative time, blood loss, operative incision, capacity draining, and time of draining are continuous variables (Sig./*p <* 0.05 was considered to be statistically significant. Exp (B) >1 were risk factors and <1 were protective factors). Italicized value is statistically different

## Discussion

This study analyzed 923 patients who underwent lumbar surgery and assessed 23 possible risk factors. Importantly, we identified new risk factors of osteoporosis and traumatic fracture. Next, we analyzed the results of our research.

### Rates of acute surgical site infections after lumbar surgery

Surgical site infections are the most common hospital-acquired infections that occur in the postoperative period [9]. However, the reported incidence of postoperative spinal infections varies widely, from 0.7 to 16% [10–13]. The reason for this wide range may be that different factors evaluated during the preoperative period have different rates of postoperative infection. Overall, however, there are relatively few reports on lumbar ASSIs. The incidence of ASSI following lumbar surgery has been shown to be lower without the use of internal fixation of the posterior spine, such as in minimally invasive surgery [14]. The rate of ASSI following lumbar surgery has been reported to be 2.6 to 3.8% [15] following internal fixation lumbar surgery. In our study, 26 of 923 patients were diagnosed with acute surgical site infection following lumbar surgery, an infection rate of 2.81%. Therefore, it is one of the key problems encountered by orthopedists and patients.

### Analysis of risk factors

Numerous factors influence the development of ASSI after lumbar surgery, and they may be divided into two categories: (1) unchangeable and strictly patient-related and (2) changeable or procedure-related.

#### Patient-related risk factors

##### BMI

When performing surgery on obese patients with a thick layer of subcutaneous fat, it is necessary to cut through a large amount of oily liquid. The sterile gauze often becomes saturated with liquefied fat from the surgical incision in obese patients, and bacteria can become embedded in the incision, increasing the risk of infection. It has been reported in the literature [14, 15] that BMI is a risk factor for postoperative complications; a 5-kg/m^2^ increase in BMI is associated with a 10% increase in the risk of postoperative complications, especially surgical site infection. In our study, we also found that a higher BMI was associated with a greater risk of ASSI. Therefore, orthopedic surgeons should assess patients’ BMI preoperatively and should be especially vigilant in caring for the surgical incisions of obese patients in the postoperative period.

##### Smoking

Tar, nicotine, and other toxic substances are absorbed into the bodies of patients with a long history of smoking. Many scholars have described the physical effects of smoking. Lin AH et al. [16] reported that reactive oxygen species in smokers will attack polyunsaturated fatty acids in the biological membranes, leading to lipid peroxidation and the formation of a large number of small-molecule lipid peroxidation products, such as malondialdehyde, acetone, and pentanaldehyde, which reflect the degree of lipid oxidative damage. Therefore, the products of lipid peroxidation can be directly or indirectly caused by injury and functional changes of cells. Finally, the surgical sites in smokers heal more slowly, and the risk of infection is increased. In our study, we found that the longer the smoking history, the greater the risk of ASSI. Therefore, orthopedic surgeons should assess patients’ smoking history preoperatively and should carefully monitor for ASSI in patients with a long smoking history.

##### Diabetes mellitus

Surgical site infections are related to the presence of diabetes mellitus. Thus, ASSI following lumbar surgery may be associated with diabetes mellitus. Patients with diabetes mellitus may have lesions in the blood vessels, including in the small vessels and the microvasculature [17]. Therefore, when the vessels are cut, large vessels and microvessels may be occluded, leading to ischemia and hypoxia in the incision tissue and, finally, to infection or a lack of healing at the lumbar surgical site. The immune function of patients with diabetes mellitus is inhibited because of serious functional damage to the cell and a decrease in platelet growth factors [18]. Therefore, the probability of acquired infection is significantly increased. Using logistic regression analysis, we also found that diabetes mellitus was associated with ASSI following lumbar surgery. Therefore, orthopedic surgeons should monitor blood glucose levels in patients preoperatively and should not perform lumbar surgery until the glucose levels return to normal with insulin or hypoglycemic agents.

##### Osteoporosis

On logistic regression analysis of 923 patients who underwent lumbar surgery, we discovered that osteoporosis was related to ASSI following lumbar surgery; no previous study has reported a relationship between osteoporosis and SSI or ASSI. Thus, the specific mechanism to explain how osteoporosis affects ASSI following lumbar surgery is unclear. Our results could be explained in two ways. There were relatively few infections in this study, which could have affected the statistical analysis results. However, we think that osteoporosis may indeed be associated with ASSI following lumbar surgery, and this is a newly discovered factor. We suppose that a vertebral body with osteoporosis cannot be firmly fixed using internal fixation, resulting in loosening of the pedicle screws. Additionally, the operation time and bleeding in patients with osteoporosis were significantly higher than in those without osteoporosis. In the future, further study is needed regarding the mechanism of osteoporosis and ASSI.

##### Traumatic fracture

Similar to osteoporosis, we found that traumatic fracture was a risk factor for ASSI following lumbar surgery in our logistic regression analysis. Few studies have reported that lumbar fracture can increase the risk of ASSI following lumbar surgery. Thus, as for osteoporosis, the specific mechanism of how traumatic fractures affect ASSI following lumbar surgery is unclear. We suppose that traumatic fractures can damage blood vessels and tissues and stimulate a bodily or local inflammatory response, leading to a large number of inflammatory factors into the blood.

#### Procedure-related risk factors

##### Use of antibiotics

According to our clinical experience and literature review, the postoperative use of antibiotics is an important measure for preventing infection. However, it is very controversial whether the use of preoperative antibiotics can reduce ASSIs following lumbar surgery. Scholars [19] hold that cleaning surgical incisions with a first- or second-generation cephalosporin can prevent bacterial infection, and surgical incision of pollution available third-generation cephalosporins. The judicial use of preoperative antibiotics may be a very useful prophylactic measure. However, some scholars believe that antibiotics should not be used before surgery, as they may produce drug resistance. Therefore, this is a very controversial issue. In our logistic regression analysis of 923 patients who underwent lumbar surgery, we discovered that the lacking of preoperative antibiotics was related to lumbar ASSI. We believe that administering a second-generation cephalosporin 30 min prior to the surgery is essential for preventing infection.

##### Type of operation

In our retrospective analysis, we discovered that the rate of infection for patients undergoing open surgery was significantly higher than that for minimally invasive surgery. Our logistic regression analysis showed open surgery to be a significant factor. Additionally, Koutsoumbelis et al. [20] stated that open surgery was not only traumatic with respect to bleeding, but there are also risks from tissue exposure to air, perhaps resulting in an increased risk of surgical site infections. We, therefore, believe that open surgery is more likely to cause ASSI than is minimally invasive surgery and that patients indicated for minimally invasive surgery should choose it when possible.

##### Operative time

Lumbar surgery requires a very meticulous and careful surgery because it involves sites around the spinal cord, so the operation time is significantly longer than that for other sites. The lengthy surgery may lead to tissue ischemia and hypoxia, with slow postoperative incision healing, resulting in an increased infection risk. Additionally, some studies [8, 21] have reported that a lumbar operation time of >3 h is associated with a significantly increased risk of postoperative infection. Moreover, many scholars believe that operative time is a risk factor for postoperative infection. Similarly, we found that operative time was related to ASSI following lumbar surgery. Therefore, surgeons should try to decrease the operation time during lumbar surgery.

##### Blood loss during the operation

Continuous bleeding during surgery not only affects the operation but also increases the operation time, while bacteria in the blood can circulate deep into the incision and increase the risk of postoperative infection. Relevant studies [22, 23] report that when the amount of blood loss is >800 ml, the risk of postoperative infection increased. In our analysis, we also showed that blood loss was a risk factor for lumbar ASSI. Increased intraoperative bleeding is primarily due to injury to the spinal canal venous plexus from fusion decompression, so the venous plexus should be carefully operated and blocked with bipolar coagulation.

##### Postoperative drainage time

Two drainage tubes are usually placed after lumbar surgery in our hospital. Therefore, we analyzed the association between drainage (duration, number of drains, and capacity) and ASSI following lumbar surgery. We found that drainage time was a risk factor for ASSI. In addition, Ando and Tamaki [24] reported that non-standardized drainage following lumbar surgery can easily lead to a deep incision infection. Ahmed et al. [25] considered that a postoperative drainage time of >72 h significantly increased the risk of ASSI. A drainage tube placed too shallow may cause deep congestion and hematoma, and one placed too deep is likely to cause infection. Therefore, we suggest that the optimal drainage tube placement time is 48 h.

### Study limitations

The limitation of this study was an insufficient case of infection, which could have led to inadequate analysis of some factors and to other risk factors not being identified. Thus, a larger sample should be used in future studies to validate these results.

## Conclusions

We discovered that BMI, smoking, diabetes mellitus, osteoporosis, traumatic fracture, open operation, operative time, operative blood loss, drainage time, and no preoperative use of antibiotics were associated with ASSIs following lumbar surgery. Despite measures intended to reduce the incidence of ASSI following lumbar surgery, they remain a common and potentially dangerous complication. Therefore, prevention is ideal, and an improved understanding of the risk factors will allow preventive measures to be improved. Surgeons should adequately analyze and evaluate risk factors in patients and then develop a prevention program. Once an infection is diagnosed, the authors recommend surgery to remove the lesion of infection, retention of the internal fixation material, and catheter drainage with a 3000-ml NaCl rinse daily for 7–12 days. Additionally, antibiotics should be used according to bacterial culture results.

## Additional file

Additional file 1: Original data of 26 patients with ASSI. (XLS 13 kb)

## Abbreviations

ASSI: Acute surgical site infections
BMI: Body mass index
COPD: Chronic obstructive pulmonary disease
